# Response of the Agile Antechinus to Habitat Edge, Configuration and Condition in Fragmented Forest

**DOI:** 10.1371/journal.pone.0027158

**Published:** 2011-11-04

**Authors:** Christopher P. Johnstone, Alan Lill, Richard D. Reina

**Affiliations:** School of Biological Sciences, Clayton Campus, Monash University, Victoria, Australia; University of Alberta, Canada

## Abstract

Habitat fragmentation and degradation seriously threaten native animal communities. We studied the response of a small marsupial, the agile antechinus *Antechinus agilis*, to several environmental variables in anthropogenically fragmented *Eucalyptus* forest in south-east Australia. Agile antechinus were captured more in microhabitats dominated by woody debris than in other microhabitats. Relative abundances of both sexes were positively correlated with fragment core area. Male and female mass-size residuals were smaller in larger fragments. A health status indicator, haemoglobin-haematocrit residuals (HHR), did not vary as a function of any environmental variable in females, but male HHR indicated better health where sites' microhabitats were dominated by shrubs, woody debris and trees other than *Eucalyptus*. Females were trapped less often in edge than interior fragment habitat and their physiological stress level, indicated by the neutrophil/lymphocyte ratio in peripheral blood, was higher where fragments had a greater proportion of edge habitat. The latter trend was potentially due to lymphopoenia resulting from stress hormone-mediated leukocyte trafficking. Using multiple indicators of population condition and health status facilitates a comprehensive examination of the effects of anthropogenic disturbances, such as habitat fragmentation and degradation, on native vertebrates. Male agile antechinus' health responded negatively to habitat degradation, whilst females responded negatively to the proportion of edge habitat. The health and condition indicators used could be employed to identify conservation strategies that would make habitat fragments less stressful for this or similar native, small mammals.

## Introduction

In studies examining habitat fragmentation and degradation effects on animals there has been a tendency to rely on distribution metrics (e.g. occurrence, abundance, density), without much reference to performance indices (e.g. litter size, survivorship, physiological stress). Fletcher et al. [1] noted that in 194 studies of fragment edge and area effects on vertebrates, distribution metrics were almost three times as common as performance indices, despite earlier authors suggesting that understanding how environmental factors limit a population or species' range requires examination of population densities and at least one index of well-being (fecundity, parasite load, body condition, growth rate etc.) [2].

Decline and extinction of vertebrate populations in fragmented habitat is variously attributed to habitat change (loss, degradation, edge effects and isolation), altered species interactions (predation, parasitism etc.), changed behaviour (edge avoidance, disrupted dispersal, social relationships or resource-tracking), altered physiology (poor body condition and chronic physiological stress) and stochastic threats associated with small population size [1], [3], [4], [5]. The area, spatial configuration, isolation and habitat degradation levels of fragments are considered the key environmental factors influencing these threatening processes [3], [6], [7], [8], [9]. However, the relative importance of the putative agents of population decline remain unclear and probably vary among taxa and landscapes [3]. Further research using diverse study areas and species is needed to properly evaluate this possibility.

We report elsewhere on performance and distribution differences between agile antechinus (*Antechinus agilis*, Family: Dasyuridae) populations living in fragmented and continuous *Eucalyptus* forest [10]. Here, we compare responses of this species to landscape configuration (e.g. fragment area, proportion of edge) and microhabitat variables [11] in an anthropogenically-fragmented landscape in order to identify possible causal relationships. The microhabitat variables were either living or dead vegetation. Abiotic features, such as rocks or human-made tracks, and features related to the presence of other animals, such as burrow entrances or dens, were never close enough to an antechinus trapping station to be recorded.

The agile antechinus is the most widespread and common native mammalian carnivore in much of our South Gippsland study area in south-east Australia [12]. It is locally common [12] and consequently not the focus of much conservation effort. However, there is a growing view in conservation biology that successful wildlife management should include a focus on common, native species, as it is preferable to prevent future decline rather than wait until such species become threatened before taking management action. The approach used here could easily be applied to other small mammals that are frequently the focus of fragmentation studies e.g. voles (subfamily *Avicolinae*) and shrews (family *Soricidae*).

We examined one distribution metric and three independent performance variables in the agile antechinus: (1) relative abundance (based on trapping rates); (2) mass/size residuals (MSR), a well-established index of fat reserves in small mammals [13]; (3) erythrocyte indicators of health status, including a novel metric, haemoglobin-haematocrit residuals (HHR) and (4) leukocyte profile indicators of hypothalamus-pituitary-adrenal (HPA) axis-mediated stress (hereinafter physiological stress [14]). This last variable encompassed the neutrophil-to-lymphocyte ratio (N∶L) and total neutrophil, lymphocyte and eosinophil concentrations in peripheral, circulating blood [15]. We used these estimates of population health status to address the following questions:

1. Are blood cell indicators of stress or health status correlated with estimated body condition (MSR)?
2. Do agile antechinus in *Eucalyptus* forest fragments use some microhabitats preferentially?
3. Are features of fragmented landscapes, such as edge habitat, fragment area, microhabitat heterogeneity etc., related to agile antechinus' abundance, body condition and blood cell indicators of stress or health status?

## Results

We captured 734 agile antechinus over 3,780 trap-nights at 30 study sites in 2007 and 2008; 165 males and 131 females were captured at fragment edges and 191 males and 247 females in the interior. Over the two study years, a subset of 263 individuals was measured for mass, morphometrics and haematological indicators of stress. Of these, 76 males and 45 females were captured in fragment edges and 71 males and 71 females in interiors. Relative abundance was calculated from capture rates for edge and interior populations (Table 1).

**Table 1:** Summary of mean (± s.e.) values obtained for stress and condition indicators in this study.

		FRAGMENTS	
Sex	Response variable	Edge (<60 m)	Interior (>60 m)
**FEMALES**	Relative abundance:	0.006±0.005	0.037±0.016
	MSR (g):	−1.05±0.62	−1.32±0.38
	HHR (g·L^−1^)^a^	−0.24±0.62	+0.15±1.78
	N∶L ratio:^b^	0.822±0.091	1.003±0.158
	Neutrophils: (×10^11^·L^−1^)	1.39±0.21	1.30±0.24
	Lymphocytes: (×10^11^·L^−1^)	1.82±0.21	1.44±0.13
	Eosinophils: (×10^9^·L^−1^)	7.19±1.76	5.64±0.94
**MALES**	Relative abundance:	0.004±0.002	0.013±0.010
	MSR (g):	+1.58±0.63	+2.13±0.70
	HHR (g·L^−1^)	−1.17±1.51	+1.28±2.03
	N∶L ratio:	0.927±0.103	0.967±0.103
	Neutrophils: (×10^11^·L^−1^)	1.42±0.20	1.38±0.14
	Lymphocytes: (×10^11^·L^−1^)	1.61±0.15	1.59±0.13
	Eosinophils: (×10^9^·L^−1^)	8.02±1.21	9.19±1.43

a HHR: Haemoglobin-haematocrit residuals.

b N∶L ratio: Neutrophil-to-lymphocyte ratio.

### Relationship between blood cell variables and body condition

In females, the model including Ht best explained variation in body condition (indexed as MSR). As an AIC difference (ΔAIC)≥2 is usually considered reasonable support for a model [16], the ΔAICs between models for female Ht and Hb can only be considered marginal (Ht-Hb ΔAIC = 2.0), whereas there is support for Ht being a better predictor of MSR (estimated fat reserves) than is HHR (Ht-HHR ΔAIC = 3.4). For males, the model including HHR best explained MSR, but the differences among models were not convincing (HHR-Hb ΔAIC = 0.9 and HHR-Ht ΔAIC = 2.1). None of the individual erythrocyte variables were significantly associated with MSR (all *P*>0.05). For subsequent analyses we use HHR as a health status indicator, as it is the most readily interpretable of the three erythrocyte variables (Tables 2, 3 and 4).

**Table 2:** Relationships between blood cell indicators of stress and health status and Mass-Size Residuals (g) for female agile antechinus.

Blood cell indicator	Variables	*df*	*t*-value	*P* a
Neutrophils	*MONTH*	25	−0.38	0.710
	Neutrophils (cells·L^−1^)	84	−0.15	0.884
Lymphocytes	*MONTH*	25	−0.42	0.681
	Lymphocytes (cells·L^−1^)	84	−0.31	0.759
Eosinophils	*MONTH*	25	−0.49	0.625
	Eosinophils (cells·L^−1^)	84	0.06	0.952
N∶L ratio^b^	*MONTH*	25	−0.65	0.519
	(log)N∶L ratio	84	0.66	0.511
Haemoglobin (Hb)	*MONTH*	25	−0.43	0.669
	Hb (g·L^−1^)	81	−1.43	0.158
Haematocrit (Ht)	*MONTH*	25	−0.28	0.784
	Ht	81	−1.96	0.054
HHR^c^	*MONTH*	25	−0.43	0.672
	HHR (g·L^−1^)	81	−0.80	0.428

a Linear mixed effect model results are shown. The *df*, *t*- and *P*-value are from restricted maximum likelihood models.

b N∶L ratio: Neutrophil-to-lymphocyte ratio.

c HHR: Haemoglobin-haematocrit residuals.

**Table 3:** Relationships between blood cell indicators of stress and health status and Mass-Size Residuals (g) for male agile antechinus.

Blood cell indicator	Variables	*df*	*t*-value	*P* a
Neutrophils	*MONTH*	26	2.18	0.039
	Neutrophils (cells·L^−1^)	112	1.14	0.257
Lymphocytes	*MONTH*	26	2.98	0.006
	Lymphocytes (cells·L^−1^)	112	2.48	0.015
Eosinophils	*MONTH*	26	2.23	0.035
	Eosinophils (cells·L^−1^)	112	2.14	0.035
N∶L ratio	*MONTH*	26	3.14	0.004
	(log)N∶L ratio	115	−0.89	0.377
Haemoglobin (Hb)	*MONTH*	26	3.25	0.003
	Hb (g·L^−1^)	105	1.15	0.251
Haematocrit (Ht)	*MONTH*	26	3.17	0.004
	Ht	105	−0.40	0.689
HHR	*MONTH*	26	3.35	0.003
	HHR (g·L^−1^)	105	1.49	0.139

a Linear mixed effect model results are shown. The *df*, *t*- and *P*-value are from restricted maximum likelihood models.

b N∶L ratio: Neutrophil-to-lymphocyte ratio.

c HHR: Haemoglobin-haematocrit residuals.

**Table 4:** Information-theoretic (AIC) parameters for blood cell measurements and time of year (*MONTH*) as explanatory models for Mass-Size Residuals (g).

Sex	Model	AIC^a^
FEMALES	Neutrophils (cells·L^−1^)+*MONTH*	491.1
	Lymphocytes (cells·L^−1^)+*MONTH*	491.0
	Eosinophils (cells·L^−1^)+*MONTH*	491.1
	(log)N∶L ratio^b^+*MONTH*	490.7
	Haemoglobin (Hb) (g·L^−1^)+*MONTH*	479.2
	Haematocrit (Ht)+*MONTH*	477.2
	HHR^c^ (g·L^−1^)+*MONTH*	480.6
MALES	Neutrophils (cells·L^−1^)+*MONTH*	789.8
	Lymphocytes (cells·L^−1^)+*MONTH*	784.9
	Eosinophils (cells·L^−1^)+*MONTH*	786.4
	(log)N∶L ratio+*MONTH*	804.2
	Haemoglobin (Hb) (g·L^−1^)+*MONTH*	745.7
	Haematocrit (Ht)+*MONTH*	746.9
	HHR (g·L^−1^)+*MONTH*	744.8

Interaction terms were examined and discarded from the models.

a Linear mixed effect model results are shown. The AIC values are from maximum likelihood models (suitable for comparing models).

b N∶L ratio: Neutrophil-to-lymphocyte ratio.

c HHR: Haemoglobin-haematocrit residuals.

There were no significant relationships between any of the female leukocyte variables and MSR. Male lymphocyte and eosinophil concentrations were significantly higher where MSR was greater (*r* = 0.20, *P* = 0.015 and *r* = 0.34, *P* = 0.036, respectively) (Tables 2, 3 and 4). However, these relationships were somewhat confounded, because all three variables were correlated with *MONTH* during the March–August trapping period (see below) and so were difficult to interpret.

### Microhabitat preference

Agile antechinus were captured more often in traps whose local microhabitat was dominated by woody debris than in traps associated with any of the other microhabitat categories (*P* = 0.033) (Table 5). No other significant relationships between capture sites and microhabitat characteristics were evident.

**Table 5:** Analysis of the difference between expected (number of traps set) and observed captures of agile antechinus as a function of microhabitat.

Microhabitat	Mean ± *SE* a	*df*	*t*-value	*P*
*DEAD EUCALYPT TREE*	+0.01±0.07	180	−0.17	0.866
*EUCALYPT* (<2 m diam.)	−0.12±0.24	180	−0.43	0.665
*EUCALYPT* (>2 m diam.)	+0.07±0.16	180	0.32	0.750
*NON-EUCALYPT TREE*	−0.17±0.10	180	0.20	0.842
*SHRUB*	−0.14±0.19	180	−0.45	0.651
*TEATREE/PAPERBARK*	−0.36±0.07	180	−1.45	0.148
*WOODY DEBRIS*	+0.48±0.27	180	2.14	0.033

a Means and *SE* are shown for residuals of number of captures (observed) and number of traps set (expected) in each microhabitat category (positive sign = greater than expected and negative sign = less than expected).

### Responses of agile antechinus to landscape configuration, edge habitat and vegetation features

#### (1) Relative abundance

Relative abundance was significantly greater for females in interior than *EDGE* habitat (*r* = 0.26, *P*<0.001). The variables with the strongest independent effects on female agile antechinus' relative abundance were *EDGE* (29.8%) and *CORE* (34.3%) (Table 6).

**Table 6:** Relationships between relative abundance and environmental variables for agile antechinus.

Sex	Explanatory variables	*r* a	*IE*	*df*	*t*-value	*P*
Females	*DI*	0.03	0.6			
	*DIST*	0.13	9.3			
	*EDGE*	0.26	**29.8**	144	5.04	<0.001
	*HETEROGEN.*	−0.02	1.6			
	*CORE*	0.27	**34.3**	27	1.91	0.066
	*MONTH*	0.05	2.7			
	*PC.1*	−0.14	9.6			
	*PC.2*	−0.16	11.1			
	*PC.3*	0.01	1			
Males	*DI*	0.1	3			
	*DIST*	0.08	7.7			
	*EDGE*	0.07	2.2			
	*HETEROGEN.*	0.14	4.9			
	*CORE*	0.31	**47.1**	24	5.19	<0.001
	*MONTH*	0.21	15.1			
	*PC.1*	−0.02	0.4			
	*PC.2*	0.19	12.2	24	2.44	0.022
	*PC.3*	0.13	7.4	24	−0.97	0.343
	*CORE×PC.3*	na	na	24	3.53	0.002

a Pearson's correlation coefficients (*r*), the independent effect of variables from hierarchical partitioning (*IE*), and results of linear mixed effect model fitting are shown. Degrees of freedom, *t*-value and *P*-values are shown for variables that were selected for inclusion in the reduced LMEM using Akaike Information Criterion.

**Figure 1:**
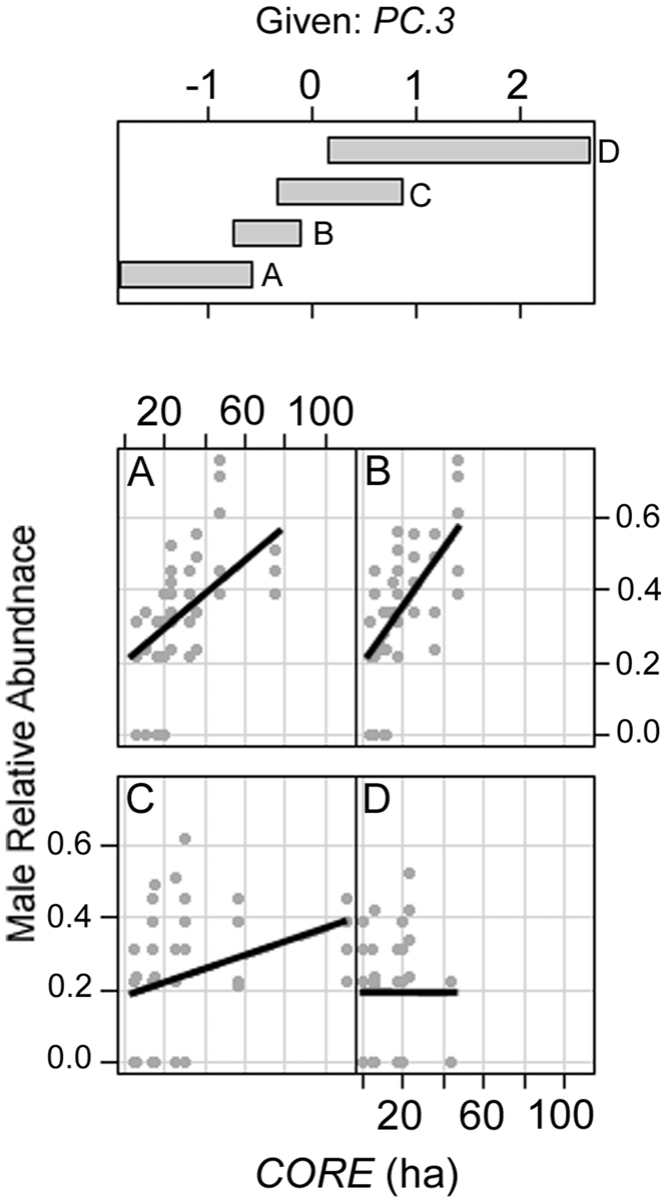
Conditioning plot of *CORE* (ha) given *PC.3* for male relative abundance. The top box shows regions of *PC.3* for which relative abundance is plotted against *CORE*. The overlap in *PC.3* is 25%. Conditioning plots show the range of a response variable (here, male relative abundance) for values of one explanatory variable (here, fragment core area, *CORE*) over given ranges of a second explanatory variable (here, vegetation condition index *PC.3*).

Male relative abundances were significantly greater where *PC.2* was higher (*r* = 0.19, *P* = 0.022), although *r* was small. Core habitat area had a significant effect on male relative abundance (*P*<0.001), but the relationship was complicated by a significant interaction with *PC.3* (*P* = 0.002). A conditioning plot of *CORE* and *PC.3* suggested that the effect of *CORE* on male relative abundance was generally positive, but that the slope of the effect was less pronounced where *PC.3* was greater (Figure 1). (i.e. male abundance was higher in larger fragments except where *PC.3* was high). The two most important variables for independently explaining male relative abundances were *CORE* (47.1%) and *MONTH* (15%) (Table 6).

#### (2) Fat stores

Estimated fat reserves (MSR) in females showed significant associations with habitat *CORE* (*r* = −0.27, *P* = 0.001), *HETEROGEN* (*r* = 0.03, *P* = 0.003), *PC.1* (*r* = 0.13, *P* = 0.008) and *PC.3* (*r* = −0.05, *P* = 0.025), although again most *r* values were small. The variables with the most important independent effects on female MSR were *CORE* (36.8%) and *HETEROGEN* (16.8%).

**Figure 2:**
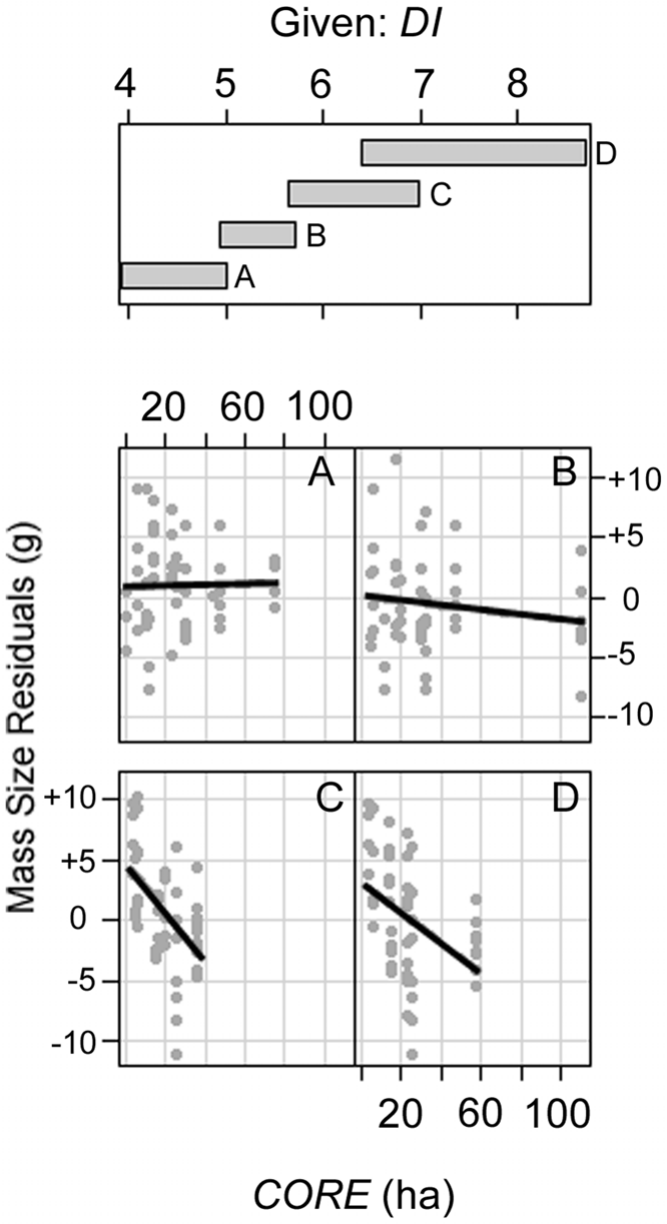
Conditioning plot of *CORE* (ha) given *DI* for male body condition index (MSR). The top box shows regions of *DI* for which MSR is plotted against *CORE*. The overlap in *DI* is 25%.

In males, fat reserves were significantly associated with fragment *DI* (*r* = −0.10, *P* = 0.034), *CORE* (*r* = −0.20, *P* = 0.037) and *MONTH* (*r* = 0.33, *P* = 0.002). The interaction term *DI*×*CORE* required interpretation before the main effects were examined (*P* = 0.059). A conditioning plot of *CORE* and *DI* suggested that the effect of the former on male MSR was generally negative, but that the slope of the effect was less pronounced where *DI* was shallower (Figure 2) (i.e. fat reserves were smaller in agile antechinus in fragments with a greater core area, but only when the fragments also had a higher ratio of edge to interior habitat). The variables that best explained variation in male fat reserves were *MONTH* (42.0%) and *CORE* (16.7%) (Table 7, Figure 2).

**Table 7:** Relationships between mass-size residuals (MSR) (g) and environmental variables for agile antechinus.

Sex	Explanatory variables	*r* a	*IE*	*df*	*t*-value	*P*
Females	*DI*	−0.19	7.5			
	*DIST*	0.1	3			
	*EDGE*	0.07	2.7			
	*HETEROGEN*	0.03	16.8	21	3.36	0.003
	*CORE*	−0.27	**36.8**	21	−3.77	0.001
	*MONTH*	−0.06	0.8			
	*PC.1*	0.13	15.2	21	2.96	0.008
	*PC.2*	−0.09	7.3	21	−2.02	0.057
	*PC.3*	−0.05	9.8	21	−2.41	0.025
Males	*DI*	−0.1	5.5	22	−2.26	0.034
	*DIST*	−0.08	3.1			
	*EDGE*	−0.08	2.5			
	*HETEROGEN.*	0.18	15.8	22	1.7	0.104
	*CORE*	−0.2	16.7	22	−2.22	0.037
	*MONTH*	0.33	**42**	22	3.46	0.002
	*PC.1*	0.04	4.5			
	*PC.2*	0.17	6.7			
	*PC.3*	0.11	3			
	*DI*×*CORE*	na	na	22	1.99	0.059

a Pearson's correlation coefficients (*r*), the independent effect of variables from hierarchical partitioning (*IE*), and results of linear mixed effect model fitting are shown. Degrees of freedom, *t*-value and *P*-values are shown for variables that were selected for inclusion in the reduced LMEM using Akaike Information Criterion.

#### (3) Haemoglobin/Haematocrit residuals

Female HHR was not significantly associated with any habitat variable. Variation in female HHR was best explained by *HETEROGEN* (27.1%) (Table 8).

**Table 8:** Relationships between haemoglobin-haemtocrit residuals (HHR) (g·L^−1^) and environmental variables for agile antechinus.

Sex	Explanatory variables	*r* a	*IE*	*df*	*t*-value	*P*
Females	*DI*	−0.1	11.5			
	*DIST*	0.04	2.7			
	*EDGE*	−0.01	0.3			
	*HETEROGEN.*	0.09	**27.1**	23	1.96	0.062
	*CORE*	0.08	9.7			
	*MONTH*	−0.09	7.3			
	*PC.1*	0.06	19.7	23	1.67	0.108
	*PC.2*	0.03	1.9			
	*PC.3*	−0.11	19.7	23	−1.63	0.116
Males	*DI*	−0.03	1.6			
	*DIST*	−0.09	10.4			
	*EDGE*	−0.08	7.3			
	*HETEROGEN.*	0.07	16.2	23	2.26	0.034
	*CORE*	0.11	10			
	*MONTH*	−0.13	14.8			
	*PC.1*	0.02	7.3	23	1.6	0.123
	*PC.2*	−0.15	19.4	23	−2.38	0.026
	*PC.3*	−0.11	12.9	23	−2.36	0.027

a Pearson's correlation coefficients (*r*), the independent effect of variables from hierarchical partitioning (*IE*), and results of linear mixed effect model fitting are shown. Degrees of freedom, *t*-value and *P*-values are shown for variables that were selected for inclusion in the reduced LMEM using Akaike Information Criterion.

In males, *HETETOGEN* (*r* = 0.07, *P* = 0.034), *PC.2* (*r* = −0.15, *P* = 0.026) and *PC.3* (*r* = −0.11, *P* = 0.027) were significantly associated with HHR, although the correlation coefficients were small. The variables that best independently explained variation in male HHR were *PC.2* (19.4%) and *HETEROGEN* (16.2%) (Table 8).

#### (4) Neutrophil-to-lymphocyte ratio

Female N∶L was strongly associated with *DI* (*r* = 0.53, *P* = 0.002). Variation in this stress index was best explained by *DI* (42.6%) and *MONTH* (25.5%). Male N∶L was strongly associated with MONTH (*r* = 0.53, *P*<0.001). For males, the best independent, explanatory variables for N∶L were *MONTH* (57.2%) and *PC.2* (19.9%) (Table 9).

**Table 9:** Relationships between (log)Neutrophil∶Lymphocyte ratio and environmental variables for agile antechinus.

Sex	Explanatory variables	*r* a	*IE*	*df*	*t*-value	*P*
Females	*DI*	0.53	**42.6**	23	3.54	0.002
	*DIST*	−0.22	4.5			
	*EDGE*	−0.18	7.8	85	−1.23	0.222
	*HETEROGEN.*	0.04	0.7			
	*CORE*	0.33	12			
	*MONTH*	0.39	**25.5**	23	1.84	0.079
	*PC.1*	−0.1	1.1			
	*PC.2*	0.13	1.7			
	*PC.3*	−0.08	4.2	23	−1.91	0.069
Males	*DI*	0.24	7.9			
	*DIST*	−0.24	7.1			
	*EDGE*	−0.06	0.9			
	*HETEROGEN.*	0.06	0.7			
	*CORE*	0.02	0.4			
	*MONTH*	0.53	**57.2**	24	4.82	<0.001
	*PC.1*	−0.14	3.9			
	*PC.2*	0.35	19.9	24	1.23	0.229
	*PC.3*	−0.04	2	24	−1.5	0.146

a Pearson's correlation coefficients (*r*), the independent effect of variables from hierarchical partitioning (*IE*), and results of linear mixed effect model fitting are shown. Degrees of freedom, *t*-value and *P*-values are shown for variables that were selected for inclusion in the reduced LMEM using Akaike Information Criterion.

#### (5) Leukocyte concentrations

In both sexes, the only significant relationship between an environmental variable and the peripheral blood neutrophil concentration was for *MONTH* (March to August) (females *r* = 0.52, *P*<0.001, independent effect = 63.0%; males *r* = 0.62, *P*<0.001, independent effect = 63.6%) (Table 10).

**Table 10:** Relationships between neutrophils (cells·L^−1^) and environmental variables for agile antechinus.

Sex	Explanatory variables	*r* a	*IE*	*df*	*t*-value	*P*
Females	*DI*	0.25	6.8			
	*DIST*	−0.01	0.4			
	*EDGE*	−0.02	0.3			
	*HETEROGEN.*	0.11	1.7			
	*CORE*	0.22	8.7			
	*MONTH*	0.52	**63**	25	4.54	<0.001
	*PC.1*	0.03	1.4			
	*PC.2*	0.22	10.3			
	*PC.3*	0.21	7.6			
Males	*DI*	0.15	1.8			
	*DIST*	−0.12	2.3			
	*EDGE*	0.02	0.4			
	*HETEROGEN.*	0.12	1.3			
	*CORE*	0.02	1.2			
	*MONTH*	0.62	**63.6**	26	5.44	<0.001
	*PC.1*	−0.06	0.8			
	*PC.2*	0.35	17			
	*PC.3*	0.25	11.7			

a Pearson's correlation coefficients (*r*), the independent effect of variables from hierarchical partitioning (*IE*), and results of linear mixed effect model fitting are shown. Degrees of freedom, *t*-value and *P*-values are shown for variables that were selected for inclusion in the reduced LMEM using Akaike Information Criterion.

**Figure 3:**
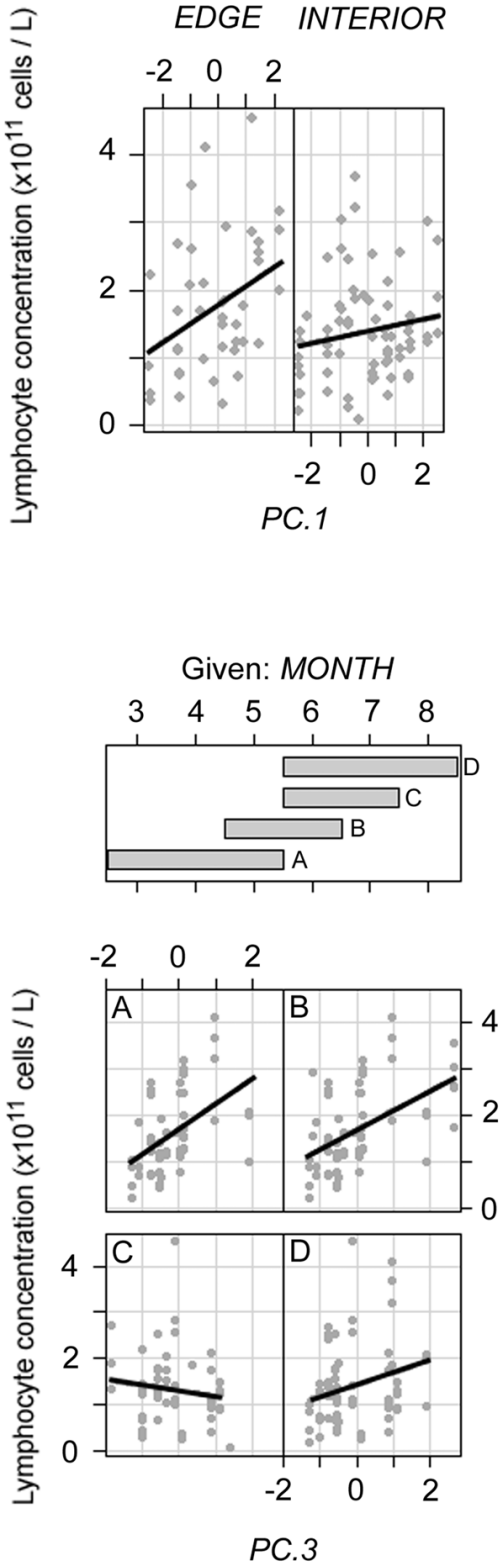
Conditioning plots of *PC.1* given *EDGE* (above) and *PC.3* given *MONTH* (below) for female lymphocyte concentration. Overlap is 25%.

In females, lymphocyte concentration was significantly associated with *DI* (*r* = −0.16, *P* = 0.008), and although *EDGE*, *MONTH*, *PC.1* and *PC.2* were included in the best model, the interaction terms *EDGE*×*PC.1* and *MONTH*×*PC.3* were also included. The relationship between *PC.1* and lymphocyte concentration was positive in both interior and edge habitat, but more pronounced in populations living near forest edges (Figure 3). The relationship between *PC.3* and lymphocyte concentration was difficult to interpret, as the correlation changed from positive to negative during the sampling period (Figure 3). The independent effects on female lymphocyte concentration were strongest for *PC.3* (22.3%) and *MONTH* (19.6%). The best explanatory variables for male lymphocyte concentration were *PC.1* (20.2%) and *PC.3* (43.3%) (Table 11).

**Table 11:** Relationships between lymphocytes (cells·L^−1^) and environmental variables for agile antechinus.

Sex	Explanatory variables	*r* a	*IE*	*df*	*t*-value	*P*
Females	*DI*	−0.16	15.7	21	−2.95	0.008
	*DIST*	0.14	2.6			
	*EDGE*	0.21	13.1	84	−2.72	0.008
	*HETEROGEN.*	−0.01	2.4			
	*CORE*	−0.07	1.5			
	*MONTH*	0.24	19.6	21	2.86	0.009
	*PC.1*	0.24	17	21	3.14	0.005
	*PC.2*	0.08	5.7			
	*PC.3*	0.29	22.3	21	−2.67	0.014
	*EDGE×PC.1*	na	na	84	−2.3	0.024
	*MONTH×PC.3*	na	na	21	3.25	0.004
Males	*DI*	−0.14	15.8			
	*DIST*	0.04	3.1			
	*EDGE*	0.08	7.1			
	*HETEROGEN.*	0.07	5.5			
	*CORE*	−0.05	2.3			
	*MONTH*	0.06	2.2			
	*PC.1*	0.14	20.2			
	*PC.2*	0	0.6			
	*PC.3*	0.24	**43.3**	26	1.89	0.07

a Pearson's correlation coefficients (*r*), the independent effect of variables from hierarchical partitioning (*IE*), and results of linear mixed effect model fitting are shown. Degrees of freedom, *t*-value and *P*-values are shown for variables that were selected for inclusion in the reduced LMEM using Akaike Information Criterion.

Neither male nor female eosinophil concentration showed a significant relationship with a potential explanatory factor, except for *MONTH* in males (*r* = 0.48, P<0.001). The variables that best independently explained variation in eosinophil concentration were *PC.3* in females (27.1%) and *MONTH* (53.9%) in males (Table 12).

**Table 12:** Relationships between eosinophils (cells·L^−1^) and environmental variables for agile antechinus.

Sex	Explanatory variables	*r* a	*IE*	*df*	*t*-value	*P*
Females	*DI*	0.08	2.8			
	*DIST*	0.22	21.2			
	*EDGE*	0.02	0.3			
	*HETEROGEN.*	0	2.9			
	*CORE*	−0.02	0.7			
	*MONTH*	0.2	15.8			
	*PC.1*	0.14	8.8	23	0.99	0.332
	*PC.2*	0.18	20.3	23	1.47	0.156
	*PC.3*	0.26	**27.1**			
	*PC.1×PC.2*	na	na	23	0.07	0.943
Males	*DI*	0.09	1.7			
	*DIST*	0.07	3.9	24	1.49	0.15
	*EDGE*	−0.13	3.6			
	*HETEROGEN.*	−0.03	1.9			
	*CORE*	−0.1	1.7			
	*MONTH*	0.48	**53.9**	24	4.11	<0.001
	*PC.1*	0.13	2.9			
	*PC.2*	0.32	19.2	24	1.3	0.206
	*PC.3*	0.24	11			

a Pearson's correlation coefficients (*r*), the independent effect of variables from hierarchical partitioning (*IE*), and results of linear mixed effect model fitting are shown. Degrees of freedom, *t*-value and *P*-values are shown for variables that were selected for inclusion in the reduced LMEM using Akaike Information Criterion.

## Discussion

### Relationships between blood cell variables and estimated fat reserves of agile antechinus

There was no convincing relationship between any immune cell variable and MSR in female agile antechinus. Male lymphocyte and eosinophil concentrations were higher when body condition indices were higher, but these associations were confounded by correlations between MSR, lymphocyte concentration and eosinophil concentration and time in the study period when trapping occurred (*MONTH*).

Haematocrit, Hb and HHR explained variation in MSR better than any of the leukocyte variables. In both sexes, HHR was positively correlated with MSR, implying that the amount of haemoglobin per unit of packed cell volume was greater in agile antechinus with larger lipid reserves. Theory and empirical evidence about chronic stress and regenerative anaemia [17], [18] both suggest that HHR is a useful index of health status in vertebrates, although it may not always be strongly related to the size of fuel stores. In vertebrates, blood loss (through parasite infection or injury), injection with stress hormones and acute stress cause elevated erythropoiesis and reticulocyte release from bone marrow [17], [19], [20]. Reticulocytes are less capable than mature erythrocytes of producing haemoglobin [21], so this process generates a blood profile in which packed cell volume may increase, but the amount of haemoglobin per unit of cell volume decreases (termed regenerative anaemia [18]).

MSR has been validated as an estimate of fat stores in several small mammals [13] but not in the agile antechinus, and an empirical evaluation of the accuracy of MSR as an estimate of lipid reserves in this species could help to clarify the relationship between HHR and MSR.

### Effects of microhabitat on capture rates

Capture rates were higher than expected where trapping station microhabitat was dominated by woody debris (logs and fallen branches), so agile antechinus could have been foraging preferentially on or beside fallen timber. Such timber could provide arthropods, such as spiders and beetle larvae, which comprise most of the study species' diet [22], as well as cover from predators [23]. Woody debris density contributed to *PC.1* (loading = −0.43), but the latter did not significantly influence agile antechinus' relative abundance in the various study sites. Thus although agile antechinus preferentially used microhabitats dominated by woody debris, fallen timber density *per se* did not affect their relative abundances at sites. In contrast, other studies [24], [25], [26] have found a positive association between *Antechinus* spp.' abundance and/or site occupancy and fallen timber volume and/or density. Fallen timber can provide nest sites [27], [28], but in our study area other factor(s) (such as predation by introduced exotics) could have kept agile antechinus' population density below a level at which nest-site availability would be limiting.

Alternatively, the positive association between woody debris and the capture rate of agile antechinus may have been caused by movement biases rather than being a direct effect of the debris on survivorship or reproductive success e.g. by non-random movement due to a preference for complex microhabitats where predation risk was lower and food abundance higher [23]. This hypothesis could be addressed by (a) trapping agile antechinus and collecting microhabitat information over larger spatial scales (i.e. the equivalent of at least several home ranges and therefore >10 ha [29]), so that the confounding effect of movement into or across trapping grids is reduced [30], or (b) radio-tracking agile antechinus and documenting their movement patterns through the fragmented landscape [31].

### Effects of fragment area on agile antechinus' relative abundance

**Figure 4:**
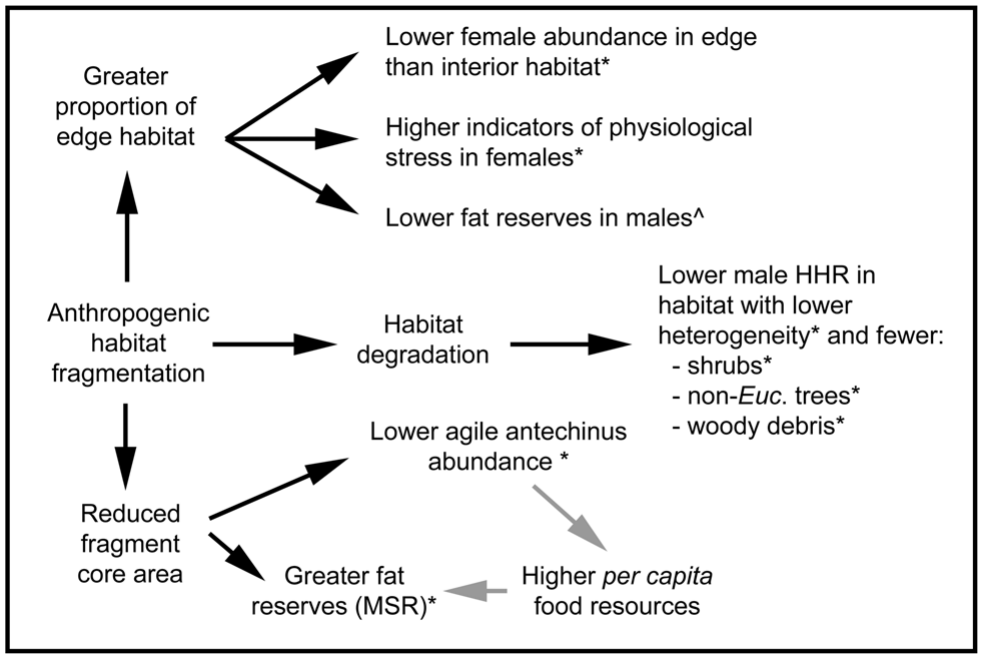
Conceptual flow diagram of the main results. There are well established associations between anthropogenic habitat fragmentation and the creation of novel edge habitat, habitat change and habitat area reduction [3]. Associations supported by significant findings are indicated by *. Findings that are significant, but may be confounded by an interaction, are indicated by ∧. Grey arrows indicate a theoretical mechanism by which an association could be operating.

Agile antechinus' relative abundance was positively associated with fragment area. Brown antechinus' population densities also vary with fragment area, but Knight and Fox [28] suggested that the relationship may have been an indirect one, in which smaller fragments were more degraded and the resultant lower habitat complexity negatively affected population density. However, in the present study the independent effect of core habitat area on relative abundance was strong (females = 34.3%; males = 47.1%) (Figure 4), suggesting that a direct effect was operating. Patch occupancy by agile antechinus in another fragmented forest was better explained by a combination of fragment area and vegetation composition than by either variable alone [32] and other investigations have reported equivocal effects of fragment size on agile antechinus' abundance [33], [34]. These varying findings could be attributable to differences in the environment (e.g. dry *vs.* wet sclerophyll forest, differences in rainfall or competitor species) or the time of year when sampling occurred.

The lower relative abundance of agile antechinus in small fragments could have been due to higher levels of predator intrusion from the agricultural matrix [35], altered emigration and/or immigration rates [36], [37], greater competition with generalist species [38], [39], [40] or reduced and/or degraded resources [28]. Theoretical models predict, and there is evidence to support the occurrence of, proportionally greater emigration from, and reduced immigration into, smaller habitat patches [1]. The rationale here is twofold, namely that dispersers are more likely to encounter large than small patches (the ‘target effect’) [36], [41], and patch-dwellers are probabilistically more likely to encounter boundaries in small than large patches, thus increasing the likelihood of emigration [37]. Given that male agile antechinus have an inherently strong propensity for dispersal [42], we might expect the effect to be stronger in males, as we observed (m = 47.1% *cf.* f = 34.3%).

### Effects of habitat structure and heterogeneity on agile antechinus' abundance and health

In both sexes of agile antechinus, *PC.1*, *PC.2* and *PC.3* had smaller independent effects on relative abundance than did *CORE*. This was surprising, given the prevailing opinion that *Antechinus* populations are strongly influenced by habitat complexity and structure [23], [25], [26], [32], [43]. The only clear support for this predominant view was that *PC.2* was positively associated with male relative abundance, although its independent effect was only 12.2% (compared with 47.1% for *CORE*). The effect of *PC.2* was that male relative abundance was higher where there were more *Eucalyptus* trees of >2 m in trunk diameter and fewer shrubs. Although large eucalypts could potentially contribute to nest-hollow, leaf litter and woody debris availability, the negative effect of shrub density on agile antechinus' relative abundance was unexpected and its cause enigmatic.

Health status of agile antechinus (indexed by HHR) was associated with certain vegetation characteristics, although the relationship was not overly convincing for females. We expect HHR to be greater in individuals in good body condition and. male HHR was higher where microhabitat heterogeneity was greater. Conceivably, heterogeneous habitat provided more foraging (and/or nesting) opportunities, so that the environment was generally less stressful. Male HHR was negatively associated with the vegetation descriptors *PC.2* and *PC.3* and *Eucalyptus* densities contributed to both of these indices. Thus males had a poorer health status in forest with denser stands of *Eucalyptus*. As capture rates of agile antechinus were higher in sites with more large eucalypts, it is plausible that there was an indirect effect of social stress or food competition on HHR when male densities were high.

Shrubs, woody debris and trees other than *Eucalyptus* contributed to *PC.2* and *PC.3*, so that a greater dominance of these microhabitat features was associated with better male health. Non-*Eucalyptus* tree species in the study area (e.g. *Cassinia* and *Olearia* spp.) frequently had fissured bark likely to harbour arthropod prey. Higher shrub density could contribute to better body condition in agile antechinus, as small mammals' foraging bouts are typically longer [44] and arthropod abundance higher where shrub cover is denser [43], although the apparent negative effects of shrub microhabitat site dominance on agile antechinus' abundance renders this interpretation necessarily tentative. Logs and fallen branches could also be promoting better health through increasing foraging resources for antechinus [28].

### Edge effects on relative abundance, stress and body condition

The trapping rate of males was not influenced by edges, but female relative abundance was significantly and markedly lower (*IE* = 29.8%) at fragment edges than in interiors. Typically, two paradigms are invoked to explain animal population distribution and abundance patterns, species sorting and habitat selection. Species sorting is characterised by random dispersal followed by non-random survivorship. Habitat selection is characterised by individual dispersal and site occupancy based on perceived rather than realised habitat quality. Species' habitat perception is a product of prior selection processes [45].

Examining the species sorting paradigm first, predation rates on birds' nests are higher in edge than interior habitat in wet *Eucalyptus* forest [46]. The same might be true for agile antechinus' tree-hollow nests, although most of Berry's [46] birds were open-cup nesters whose nestlings were probably inherently more vulnerable than concealed antechinus young. However, if fewer dependent young survive at the edge than in the interior of fragments, over several generations this could lead to successively fewer females living in edge habitat because females normally remain in the natal home range throughout life [42], [47]. Different predation rates could help explain the observed population differences, but could other factors be playing a role? Caughley et al.'s model [2] proposes that population density and condition measurements along an ecological gradient from core to peripheral habitat can be used to infer what processes are regulating populations at the edge of their range. According to Caughley et al's model the most likely explanation is that female range at forest edges was limited by a resource that was used consumptively (e.g. limited food) or pre-emptively (e.g. nest hollows). In contrast, Caughley's model suggests that male range at forest edges was limited by a change in substrate or environmental factor that was not alterable by the study species. As the forest fragments in this study had sharp forest-field boundaries, perhaps male range was simply limited by the extent of canopy cover?

With respect to the habitat selection paradigm, the sex difference in relative abundance in fragment edges could be related to the species' lek breeding system. Spatial segregation of the sexes outside the breeding season is well-known in lek-breeding mammals [48], [49]. The predation risk hypothesis [49], [50] predicts that to maximize their fitness, females in lekking species should make more use of habitat with a lower predation-risk, whereas males should use habitat with more abundant foraging resources, even if predation-risk is also higher there. The rationale is that males in good condition can produce many more young than good condition females, so the potential fitness benefits of ‘riskier’ foraging are different for the sexes [49]. Forest-field ecotones are more resource-rich than forest interiors and invertebrate species richness generally declines with distance from edges in forests [51]; however, edge habitats also have higher nest predation rates, at least in birds, which may indicate greater predator activity in general [46], [51], [52]. At least one other study has also reported that female agile antechinus may generally occupy better quality habitat than males [32].

The hotspot theory of lek sitting [53] predicts that during the breeding season, males should aggregate where female traffic is greatest. Male brown and agile antechinus can move large distances during or before the breeding season [54], [55]; it would be interesting to determine whether males living in edge habitat move into the fragment interior where female population density is higher immediately prior to, or at this time. Equally, females might forage nearer edges during lactation when metabolic demands are high, at least until young detach from the pouch (∼5 weeks post-parturition) when the need to return to the nest to nurse them could restrict this behaviour [55].

Female N∶L was significantly higher in fragments with a large proportion of edge habitat. Assuming that N∶L was a positive index of stress, this finding implied that female agile antechinus found such fragments more stressful than those with relatively more interior habitat. This might not be an effect of edges *per se*; if females avoided edge habitat, limited availability of core habitat in more dissected fragments could have resulted in crowding, psychosocial stress and competition for nest sites and food in the interior.

### Effects of environmental features on stored lipid reserves

Mass-size residuals, an estimate of stored fat reserves, allow us to make some inferences about whether *per capita* food resources varied among fragments. The most convincing significant relationship between MSR and a landscape variable was the negative association between MSR and core habitat area i.e. in larger fragments, the estimated stored lipid reserves were smaller. The association was strong in both sexes (females = 36.8%; males = 42.0%). Therefore nutritional stress was almost certainly not a factor causing the lower relative abundance of agile antechinus in smaller forest fragments. One possible explanation for this situation was that inter- and/or intra-specific competition for food was more pronounced in larger fragments. Experimental food provisioning suggests that inter-specific competition between agile antechinus and bush rats can be intense [39] and the latter were present in many of our study sites. Agile antechinus' relative abundance was also greater in larger fragments, so intra-specific competition may also have contributed to the observed *CORE*×MSR relationship.

### Environmental features affecting immune cell variables

Female N∶L was influenced by the proportion of edge habitat in a fragment, but male N∶L was not convincingly correlated in a consistent manner with landscape configuration, proximity to forest edge or the vegetation composition indices (*PC.1*, *PC.2*, *PC3*).

Absolute leukocyte concentrations in peripheral blood can be more informative of population health status than N∶L alone [56]. Neutrophil concentrations in both sexes were unaffected by any measured environmental variable, but they responded strongly to seasonality (females = 63.0%; males = 63.6%) i.e. concentrations increased during the March (post-dispersal) to August (pre-breeding season) sampling period. Numerical domination of neutrophils in peripheral blood may reflect greater innate immunocompetence [57], [58] and presumably the neutrophilia in agile antechinus later in the sampling period (July–August) resulted from neutrophil trafficking, production or release from bone marrow. This might constitute a form of ‘preparation’ for breeding and the synchronized breeding rut, during which physical contact among individuals, and hence the risk of disease transmission, probably increased.

Female lymphocyte concentrations responded to a broad set of environmental variables, including interactions between *EDGE* and *PC.1* and *MONTH* and *PC.3*. However, the only unambiguous relationship was that with the proportion of edge habitat in a fragment (*IE* = 15.7%). Trafficking of lymphocytes away from peripheral blood into the skin, lymph nodes and spleen, where they are more likely to be useful in the event of injury, is the most frequently cited mechanism underlying the increased N∶L observed in chronically-stressed vertebrates [15], [59], [60], [61]. Thus it appears likely that lymphopoenia produced the positive association between N∶L and edge habitat in female agile antechinus.

Eosinophil concentrations were not convincingly related to any environmental variable. In males, they were higher nearer to the breeding season. As eosinophils are strongly associated with defence against metazoan infections [62], this increase could be a ‘preparatory’ mechanism similar to the neutrophilia discussed above.

### What was limiting agile antechinus populations?

The conceptual summary of the demographic, physiological and population health findings of this study in Figure 4 could be used to predict the response of *Antechinus* spp. or other small mammals to anthropogenic habitat fragmentation There were independent and differing effects of a small habitat area, a greater proportion of edge habitat and microhabitat change in anthropogenically fragmented environments. Fragments with more edge and/or more degraded microhabitats affected population health indicators (MSR, N∶L and HHR) negatively, whereas a smaller core area reduced population abundance. Potentially, populations inhabiting small fragments that exert greater edge effects and/or are more degraded experience the interactive effects of reduced population size and lowered body condition [63], making their conservation problematic.

In the present study, agile antechinus' relative abundance decreased from core (larger, unsubdivided, forest fragments) to peripheral habitat (smaller fragments), whereas MSR was either constant (females) or increased (males) along such a gradient (Figure 4). From this dichotomy, Caughley et al.'s [2] model would suggest that the population limiting factor is probably a resource used consumptively or pre-emptively, which could be nesting sites for agile antechinus in small fragments. The model argues that if predation, disease or parasites are regulating a population, body condition should decline at the periphery of its range where the environment is more stressful. In fragments with a large proportion of edge, male MSR and female N∶L decreased from interior to edge, so conceivably at forest edges such factors were limiting population density. Thus two regulating factors could be co-occurring: 1) limited nest-site availability in smaller fragments and 2) higher rates of predation or disease in edge habitat.

It is difficult to unravel the interacting effects of fragment area and proportion of edge. On balance, the simplest explanation is that predation rates were higher in fragments with more edge habitat and predation was holding population levels below that at which *per capita* food availability would limit population size. European red foxes (*Vulpes vulpes*) and feral cats (*Felis catus*) were present and do prey upon *Antechinus* spp [64], [65]. If agile antechinus living near fragment edges were more exposed to such predators, it would help to explain why females apparently found forest edges more stressful than interiors.

### Conservation implications

For the conservation management of agile antechinus in the study area, we suggest that preserving forest fragments with large core areas, a high level of microhabitat heterogeneity and a minimum of edge habitat would help to mitigate the negative effects of habitat fragmentation on this species. This conclusion is in accordance with theories of how anthropogenic habitat fragmentation affects native vertebrates [3], although we could only identify negative effects of habitat area reduction, increased patch dissection and lower microhabitat heterogeneity by examining relative abundance and multiple performance metrics.

## Materials and Methods

### Study area and design

Research involving live animals followed the guidelines approved by the American Society of Mammalogists [66] and was conducted in accordance with local animal ethics legislation. Trapping and data collection were conducted under Monash University Biological Sciences Animal Ethics Committee permits BSCI/2008/03 and BSCI/2006/05 and the Victorian Department of Sustainability and Environment permit 10003798. Effort was made to minimize suffering and stress experienced by animals during trapping and handling.

**Figure 5:**
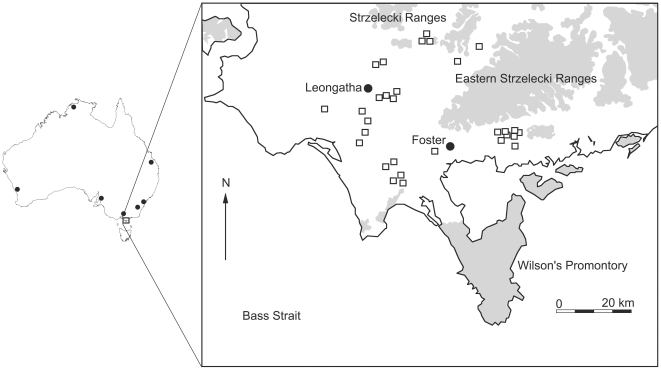
Study region in South Gippsland, south-east Australia. White = cleared agricultural land. Shaded areas = native tree cover (includes native regrowth, old growth forest and native plantations). Approximate locations of fragment study sites are indicated by white boxes (**□**). Map based on DSE interactive forest-explorer online maps (‘Forest-Explorer Online’ maps, http://www.dse.vic.gov.au).

This study was conducted from April to August 2007 and March to August 2008 in South Gippsland, Victoria, Australia (Figure 5). We sampled thirty *Eucalyptus* forest fragments dispersed in an anthropogenically-disturbed, agricultural landscape in an area bounded by the coordinates 38°35′25″S 145°41′41″E, 38°21′55″S 146°06′10″E, 38°37′19″S 146°28′20″E and 38°45′12″S 146°01′33″E. The fragments, 4.8 to 293.6 ha in area, were situated 2.1 to 38.6 km from any area of continuous forest (defined as >1000 ha of continuous, native treecover, Figure 5). Habitat similarity among study sites was achieved by restricting sites to forest stands composed of the three Ecological Vegetation Classes (EVC) [67] ‘Lowland Forest’, ‘Wet or Damp Forests (Wet)’ and ‘Wet or Damp Forests (Damp)’. Most sites contained a mixture of the first two, but some also contained small areas of the EVCs ‘Riparian Forests or Woodlands’ or ‘Rainforests’.

### Study Species

The agile antechinus is a scansorial, nocturnal marsupial restricted to south-eastern Australia. Its diet comprises terrestrial invertebrates, supplemented by some small vertebrates and scavenging from carcasses [22]. Home range area can be up to 5 ha, but is more typically 1–3 ha [29], [55]. Pre-1998 this species was considered part of the brown antechinus (*A. stuartii*) species-complex [68]; the two species have very similar life-histories and morphology [12] and authors frequently cite studies of one when discussing theories about the other.

*Antechinus* are unusual because they are semelparous [69]. A synchronized breeding rut in the Austral winter (in August in our study area) is followed by senescence and death of all males. During the 2–3 week breeding season, male foraging behaviour is reduced and lek behaviour occurs, apparently involving extended periods of male ‘vigilance’ in tree-hollow nests [70], [71]. A negative nitrogen balance develops in males, which are eventually unable to obtain sufficient food for self-maintenance [72]. Sperm storage in females, relatively protracted oestrus (≤21 days in captivity) and promiscuous mating behaviour by both sexes generate a high level of intra-sexual competition among males, with larger individuals and those that mate closer to the time of a female's ovulation typically siring more young [70], [73], [74]. After the weaning of young, a male-biased dispersal occurs in the Austral late summer [47] (January–February in the study area). Most females die after weaning their only litter [75], although a few breed in a second year (∼15% in *A. stuartii* [76]).

### Trapping protocols

Live-traps were baited with a mixture of rolled oats, peanut butter, water and vanillin. Sufficient bait was supplied for trapped agile antechinus and by-catch small mammals to eat *ad libitum*. To reduce the risk of animal death during trapping due to stress or inclement weather, traps were weather-proofed with a plastic bag and provided with bedding and a plastic refuge tube. They were set no earlier than 3 h before dusk and checked no later than 3 h after dawn.

Ten small (<30 ha), ten medium (30–60 ha) and ten large (60–300 ha) fragments were used and they were allocated randomly to a sequence for trapping. However, the fragment size categories were not used in data analysis because including the actual area of fragments as a covariate probably generated more accurate results. One trapping grid in each fragment was placed in edge (<60 m from the forest-field ecotone) and one in interior habitat (always >80 m, often 200–400 m and sometimes up to 500 m from the ecotone). The two trapping grids had 21 traps each, arranged in three lines of seven (i.e. 21 traps in 4800 m^2^). Trapping was conducted for three successive nights in each fragment. We considered captures per trap-night to represent agile antechinus' relative abundance and used this as an estimate of population density.

### Lipid reserve estimation and haematological methods

All captured individuals were sexed by visual inspection. On each trapping day, the first two ‘new’ (i.e. not previously captured) agile antechinus captured at the edge and the first two in the interior of a fragment were measured to determine total and differential leukocyte counts, mass (±0.1 g) and linear distance from nose to vent (NV) (±0.1 mm). Only single morphometric measurements were taken, which is not ideal [77], but this was unavoidable; individuals were already subjected to prolonged handling during blood sampling such that the additional handling required for multiple measurements would likely have unreasonably stressed individuals. A <1 mm disc of pinna tissue was removed from a unique position to facilitate identification on recapture to ensure that recaptured individuals were not re-sampled.

Blood-sampling was conducted within 15 min of removing an antechinus from a trap. Blood was collected before measuring the animal's mass and size to reduce the potentially confounding effects of handling stress and consequent leukocyte trafficking on leukocyte counts [60], [61]. Blood volume collected never exceeded 100 µL (∼0.1 g) and so was unlikely to have markedly affected subsequent measurement of mass. The possibility that trapping and/or handling stress could have influenced leukocyte measurements [78] is addressed in the Discussion.

Blood samples were collected by capillarity in heparinised microhematocrit tubes after puncturing one of the two lateral veins near the base of the tail with a 27 gauge needle. Whole blood haemoglobin concentration (Hb) (±0.1 grams per litre [g•L^−1^]) was determined immediately with a Hemocue 201+ haemoglobinometer (Hemocue®, Ängelholm, Sweden). All other blood samples were stored on ice and processed within 10 h, and no deterioration was observed. Haematocrit (Ht) (±0.1 mm) (%) was determined by centrifugation for 3 min at 12,700 g. Hb and Ht alone are potentially difficult to interpret, as high and low values can be caused by several factors (e.g. anaemia, dehydration, disease) [21]. Therefore we derived an index of health status based on Hb/Ht residuals, based on a similar principle to that used for deriving MSR (see Discussion).

Blood smears for differential leukocyte counts were made by the pull-wedge method [21] and stained with May-Grünwald-Geimsa stain [21]. Counts were obtained by making visual sweeps from the ‘head’ to the ‘tail’ of each smear under 400× magnification. They comprised >200 leukocytes and were all conducted by the same person. Population mean neutrophil/lymphocyte ratios were calculated from mean proportions of neutrophils and lymphocytes, as averaging ratios can generate spurious results [79]. To make total white blood cell counts (WBC), 5 µL of blood were diluted with Natt and Herrick's solution at a ratio of 1∶200 [80]. Counting was conducted under 400× magnification using an improved Neubauer haemocytometer (Blau Brand, Germany). Total neutrophil, lymphocyte and eosinophil concentrations were derived from total and differential leukocyte counts.

Mass-size residuals were derived by calculating the residuals of body mass as a factor of NV. Ordinary least squares (OLS) regressions were used to generate HHR and MSR [13].

### Relationship of haematotogical variables to body condition

We used linear mixed effect models (LMEM) to examine whether erythrocyte variables, neutrophil, lymphocyte and eosinophil concentrations or N∶L ratio were significantly related to the measured body condition index, MSR. In all LMEM, the factor *SITE* (i.e. each fragment) was included as a random effect to avoid pseudoreplication and the covariate *MONTH* (March = 3 to August = 8) was included because there are biological reasons to expect some variation in body condition to be explained by the time of year [81]. Final models were validated graphically using ordinary and standardized residuals [82].

### Handling and trapping stress

Stress indices, such as N∶L, can alter sufficiently rapidly to potentially be confounded as baseline measures by the effect of trapping and sometimes even of handling [78], [83]. However, this is *not* true of erythrocyte variables (e.g. HHR), in which it can take as long as 48 h before a peak response to an acute stressor occurs [84]. We eliminated the possibility that handling stress affected N∶L through a validation trial in which agile antechinus were blood-sampled 0, 10, 20 and 30 min post-removal from a trap [85]. Detecting trapping stress requires the immediate killing of trapped animals to establish baseline values for each study site [78], an impractical and ethically contentious procedure in our investigation.

Arguably the most appropriate interpretation of N∶L in the present study is that it reflected an additive or multiplicative response [86], [87], [88] to a combination of environmental and trapping stress. We have no reason to think that time spent in traps differed among sites. Moreover, trapping evoked a stress response in meadow voles (*Microtus pennsylvanicus*) [78], but its magnitude did not increase as a function of time spent in the trap (i.e. trapping could be considered a uniform stressor). Therefore probably the most accurate interpretation here is to view N∶L as a positive index of stress [15] and assume that significant differences in this ratio among sites are more likely due to differences in background environmental stress than in mean duration of trap occupancy. However, stress responses have not been widely studied in free-living, small mammals, so unexpected interactions of chronic and acute stress could occur [89], and therefore the interpretation of N∶L presented here is necessarily tentative.

### Response of agile antechinus to microhabitat, vegetation features and landscape configuration

We documented the dominant local microhabitat in a 3 m radius around each trapping station, using a system of 48 categories. The categories were devised during preliminary fieldwork to record as much variation in microhabitats as possible, but for analysis we used variable reduction methods (principal component analysis, PCA, and model simplification [90]) to reduce the number of categories to a manageable seven for analysis and interpretation (Table 13). More details of these categories can be obtained from the corresponding author on request. We derived residuals of trap-nights conducted in a given microhabitat (expected) and number of captures (observed) for each microhabitat for each trapping grid (i.e. from a linear model in which captures in microhabitats was treated as a function of trap-nights in microhabitats).

**Table 13:** Eigenvalues and component loadings from principal component analysis of simplified microhabitat variables.

		*PC.1*	*PC.2*	*PC.3*
	**Eigenvalues**	1.55	1.47	1.20
**Component loadings** a				
*DEAD EUCALYPT TREE*		−0.17	0.12	**0.76**
*EUCALYPT* (<2 m diam.)		**0.48**	0.34	−0.36
*EUCALYPT* (>2 m diam.)		−0.39	**0.41**	−0.23
*NON-EUCALYPT TREE*		**−0.49**	0.31	−0.29
*SHRUB*		0.13	**−0.63**	−0.18
*TEATREE/PAPERBARK*		0.38	0.37	0.36
*WOODY DEBRIS*		**−0.43**	−0.26	0.10

a Principal component axes 1–3 of 7 are shown. The dominant microhabitat was recorded at each trapping station as one of 48 microhabitat categories. The categories shown here are simplifications of the field categories derived by a model simplification procedure. Trap station microhabitats were treated as pseudo-random samples of the microhabitats in each study site. Bold values are component loadings >+/−0.40.

Trapping stations represented pseudo-random samples of microhabitat. Therefore we constructed vegetation feature indices by applying a PCA to station microhabitat feature occurrences (Table 13). We used PCA axes 1, 2 and 3 (*PC.1*, .*2* & .*3*), which had Eigenvalues>1, as vegetation descriptors, and these were included as explanatory variables in linear models examining indices of agile antechinus' stress and condition. We were also interested in whether habitat heterogeneity influenced agile antechinus population health, and so used the 48 original microhabitat categories to derive a Shannon's heterogeneity index for each site [91] which served as a habitat complexity index (*HETEROGEN*) in analysis.

We use the term ‘configuration’ to encompass fragment area and spatial configuration (shape and degree of isolation). Fragment configuration data were obtained from online native vegetation cover maps (1∶ 75,000) from the Victorian Department of Sustainability and Environment (‘Forest-Explorer Online’ maps, http://www.dse.vic.gov.au), estimated using ImageJ (http://rsbweb.nih.gov/ij/) (measured in pixels and converted to appropriate units). We measured the following fragment variables: (a) largest inside circle (*CORE*, ha), (b) ‘nearest neighbour’, the distance (m) to the nearest *Eucalyptus* fragment of >5 ha (*DIST*) and (c) dissection index (*DI*). *CORE* is an estimate of unsubdivided fragment area, which we term ‘core area’. Dissection Index was estimated by taking the ratio of the perimeter (P) of the fragment to the square root of its area (A) and scaling the results, so that for a circle *DI* = 1.0 and values >1.0 are increasingly dissected: *DI* = P/(2·(√ (π·A)) [92].

### Data analysis

The responses of the sexes to habitat fragmentation were analysed separately, as the behaviour, morphology and physiology of male and female *Antechinus* differ markedly [81], [93], [94]. All data were analysed with R 2.11.1 [95] (packages ‘nlme’, ‘MuMIN’ and ‘hier.part’) and checked for normality and homoscedasticity. Relative abundance (RA; captures per trap-night) was square-root arcsine-transformed and N∶L was log_10-_transformed to achieve normality where appropriate, but no other transformations were needed.

Linear mixed-effects models (using maximum likelihood) were applied to explanatory factors (*EDGE* response: edge or interior and *MONTH*) and covariates (*DI*, *DIST*, *CORE*, *PC.1, 2, 3* and *HETEROGEN*), and to response variables (RA, MSR, HHR and differential leukocyte parameters of stress) for all subsets model selection using the Akaike Information Criterion (AIC) (‘dredge’ in ‘MuMIN’). We checked for correlation structures in the data (sphericity, auto-regression etc.) and included these in the final model where warranted. Restricted maximum likelihood (REML) was used to generate the final models.

Where interactions among factors occur in linear models, they must be interpreted first [96], one consequence of which is that the main effects are not always interpretable. We used conditioning plots to examine interactions, but provide only provisional interpretations. We used hierarchical partitioning [97] to help infer the relative percentage of variation in each response variable that was explained by each predictor variable. In this procedure, if a variable has a total influence of 50% it indicates that it explained 50% of the variation explained by the cohort of explanatory variables used, not 50% of the total variation in the response variable. We report the independent effect (*IE*) of explanatory variables and consider variables with *IE*>25% to have had a potentially important influence on the response variable in question.
